# Extracellular proteases are key mediators of *Staphylococcus aureus* virulence via the global modulation of virulence-determinant stability

**DOI:** 10.1002/mbo3.55

**Published:** 2012-12-11

**Authors:** Stacey L Kolar, J Antonio Ibarra, Frances E Rivera, Joe M Mootz, Jessica E Davenport, Stanley M Stevens, Alexander R Horswill, Lindsey N Shaw

**Affiliations:** 1 Department of Cell Biology, Microbiology & Molecular Biology, University of South FloridaTampa, FL; 2 Department of Microbiology, Roy J. and Lucille A. Carver College of Medicine, University of IowaIowa, IA

**Keywords:** Pathogenesis, protease, proteolysis, virulence determinant

## Abstract

*Staphylococcus aureus* is a highly virulent and successful pathogen that causes a diverse array of diseases. Recently, an increase of severe infections in healthy subjects has been observed, caused by community-associated methicillin-resistant *S. aureus* (CA-MRSA). The reason for enhanced CA-MRSA virulence is unclear; however, work suggests that it results from hypersecretion of *agr*-regulated toxins, including secreted proteases. In this study, we explore the contribution of exo-proteases to CA-MRSA pathogenesis using a mutant lacking all 10 enzymes. We show that they are required for growth in peptide-rich environments, serum, in the presence of antimicrobial peptides (AMPs), and in human blood. We also reveal that extracellular proteases are important for resisting phagocytosis by human leukocytes. Using murine infection models, we reveal contrasting roles for the proteases in morbidity and mortality. Upon exo-protease deletion, we observed decreases in abscess formation, and impairment during organ invasion. In contrast, we observed hypervirulence of the protease-null strain in the context of mortality. This dichotomy is explained by proteomic analyses, which demonstrates exo-proteases to be key mediators of virulence-determinant stability. Specifically, increased abundance of both secreted (e.g. α-toxin, Psms, LukAB, LukE, PVL, Sbi, γ-hemolysin) and surface-associated (e.g. ClfA+B, FnbA+B, IsdA, Spa) proteins was observed upon protease deletion. Collectively, our findings provide a unique insight into the progression of CA-MRSA infections, and the role of secreted proteolytic enzymes.

## Introduction

*Staphylococcus aureus* is a highly successful and diverse pathogen causing an array of diseases. *S. aureus* infections typically proceed from localized sites (e.g. wound), and can proliferate via bacteremia to life-threatening systemic diseases, such as osteomyelitis, endocarditis, and septic arthritis. This diversity and pathogenic success can be attributed, largely, to its vast array of temporally and environmentally regulated virulence factors (Lowy 1998; Novick 2006). Formerly, *S. aureus* infections were confined to healthcare settings, afflicting the immunocompromised and elderly. Recently, there has been a shift in *S. aureus* epidemiology, with increased incidences of severe invasive disease in healthy subjects lacking predisposing factors (Moran et al. 2006; Johnson et al. 2007). This trendshift is the result of emerging, hypervirulent strains of methicillin-resistant *S. aureus* (MRSA) that have evolved within the community (CA-MRSA). Of considerable concern, these CA-MRSA strains appear to be moving into clinical settings and displacing existing hospital-associated MRSA isolates (Popovich et al. 2008; D'Agata et al. 2009; Webb et al. 2009).

Several CA-MRSA lineages have appeared in the last decade (McDougal et al. 2003; Diep and Otto 2008; Limbago et al. 2009), with USA300 now representing the major clone in the U.S. (Tenover et al. 2008). The reason for the surprising success of this strain as the primary CA-MRSA, and perhaps MRSA, isolate is somewhat unclear; however, work by a number of groups suggests it may be attributable to the differential expression of core genomic elements (Li et al. 2009), including the PSMs, hemolysins, enterotoxins, and extracellular proteases (Adem et al. 2005; Wang et al. 2007; Diep and Otto 2008; Montgomery et al. 2008; Kobayashi and DeLeo 2009; Li et al. 2009). With regards to this latter class of enzymes, *S. aureus* possesses 10 major secreted proteolytic enzymes. These include a metalloprotease (aureolysin, *aur*), a V8 or SspA serine protease, two cysteine proteases (staphopain A (ScpA) and staphopain B (SspB)), and six serine-like proteases that are SspA homologues (SplABCDEF) (Reed et al. 2001; Shaw et al. 2004).

A number of studies have been conducted to determine the contribution of extracellular proteases to the disease process; however, many have been contradictory. In the RN6390 background, an SspA mutant displayed attenuated virulence in three different animal models of infection (Coulter et al. 1998). Similarly, *sspABC* and *sspBC* mutations in strain 8325-4 also showed reduced virulence in a murine skin abscess model (Shaw et al. 2004). In addition to these findings, it has been shown that both cysteine proteases induce vascular leakage and shock in a guinea pig model of infection (Imamura et al. 2005). Furthermore, it was shown that the ability of Newman to evade killing by primary human macrophages is dependent on a functional aureolysin gene (Burlak et al. 2007; Kubica et al. 2008). Finally, a number of studies have shown that Aur, SspA, and SspB are produced upon engulfment by human neutrophils, and that antibodies are generated against these enzymes during infection (Burlak et al. 2007; Calander et al. 2008; Holtfreter et al. 2009). In contrast, several other studies have produced opposing results regarding the pathogenic role of extracellular proteases. Specifically, single mutations in *aur* and *scpAB* using strain 8325-4 had no effect on skin abscess formation (Shaw et al. 2004), whereas a nonpolar *sspA* mutant in RN6390 displayed enhanced virulence in a similar model (Rice et al. 2001). Additionally, mutants in *sspABC*, *sspB, aur*, and *scpAB*, in the SH1000 background produced no attenuation in virulence in a murine-septic arthritis model (Calander et al. 2004). It was also observed that a *splABCDEF* deletion mutant showed no significant difference in virulence in a murine peritonitis infection model (Reed et al. 2001).

In addition to these more general functions, *S. aureus* exoproteases have been shown to cleave specific host proteins. Staphopain B can degrade human fibronectin, fibrinogen, and kininogen, and may contribute to the ability of *S. aureus* to disseminate (Massimi et al. 2002; Imamura et al. 2005). Secreted proteases can also cleave human α1-proteinase inhibitor (Potempa et al. 1986), the heavy chains of all human immunoglobulin classes (Prokesova et al. 1992), and elastin (Potempa et al. 1988), which aids in tissue invasion. Beyond their interaction with the host, it has been demonstrated that secreted proteases modulate the stability of self-derived virulence determinants. Specifically, SspA was shown to cleave surface proteins, including fibrinogen-binding protein (McGavin et al. 1997) and surface protein A (Karlsson et al. 2001). In addition, Aur cleaves the surface-associated protein clumping factor B (McAleese et al. 2001). Cleavage of these proteins by extracellular proteases is thought to affect the transition from an adhesive to an invasive phenotype. It has also been suggested that extracellular proteases can cleave secreted toxins in order to regulate the abundance of virulence factors depending on the specific niche encountered within the host (Lindsay and Foster 1999). Indeed, it has recently shown that aureolysin modulates the stability of both α-toxin and phenol-soluble modulins in CA-MRSA strains (Zielinska et al. 2011; Gonzalez et al. 2012).

Consequently, while there is a wealth of information on the role of secreted proteases in *S. aureus* disease causation, the specific role of these enzymes as virulence factors remains unclear. Therefore, in this study, we sought to define the collective impact of this class of enzymes on pathogenesis and virulence-determinant stability. This was achieved using the CA-MRSA strain USA300, which is known to hyperproduce secreted proteases, and a strain genetically lacking all 10 of these enzymes.

## Experimental Procedures

### Bacterial strains, plasmids, and growth conditions

The CA-MRSA USA300 LAC isolate AH1263 served as the wild-type strain for analysis in this study. A derivative of this has been generated and described previously (Wormann et al. 2011) that lacks all 10 major secreted proteases (strain AH1919). This was generated as follows: Plasmids for the silent deletion of *sspAB* and *scpA* were prepared using the pKOR1 derivative, pJB38 (Wormann et al. 2011). Next, a USA300 LAC aureolysin mutant was generated using a previously made pKOR1::*aur* silent-deletion construct (Kavanaugh et al. 2007). Once confirmed, this strain was used for the subsequent inactivation of *sspAB*, followed by *scpA*, using the pJB38 plasmids referred to above, and a protocol established for pKOR1 gene inactivation (Bae and Schneewind 2006). Finally, an existing Δ*splABCDEF::erm* (Δ*spl::erm*) mutation (Reed et al. 2001) was transduced into this strain using bacteriophage 80α to create a LAC Δ*aur*Δ*sspAB* Δ*scpA* Δ*spl::erm* total exoprotease deletion strain.

Strains were grown in TSB as documented (Shaw et al. 2008), using the following protocol: 1 mL of overnight *S. aureus* cultures were used to inoculate fresh medium and allowed to grow for 3 h. These exponentially growing cultures were used to seed new medium at an OD_600_ of 0.05. These exponentially growing test cultures were then allowed to grow for the necessary time periods. Growth in milk broth was performed as previously described (Carroll et al. 2012). Briefly, exponentially growing cultures of the LAC wild-type and protease-null strain were washed three times with PBS and resuspended in 100 mL 10% skimmed milk. The initial inoculum of each strain was also determined at this time by serial dilution and plating on TSA. Cultures were incubated at 37°C with shaking and the cfu/mL of each strain determined at the times indicated, again by serial dilution and plating. For growth in pig serum, exponentially growing cultures of the LAC wild-type and protease-null strain were washed three times with PBS and resuspended in 1 mL of pig serum (Sigma). In each case, approximately 1 × 10^6^ cfu/mL of exponentially growing cells were inoculated for each strain. Cultures were incubated at 37°C with shaking and the cfu/mL of each strain determined by serial dilution and plating, every hour for 5 h. The initial inoculum of each strain was also determined from the original culture in the same manner. Data are presented as percentage survival of each strain compared with initial inocula. All growth experiments represent at least three independent biological replicates. Statistical significance was evaluated using a Student's *t*-test.

### Real-time PCR

Quantitative real-time PCR analysis was conducted as described previously (Miller et al. 2012) using primers specific for RNAII (F- ATGCGCTGATGATATACCACG, R- GTTGATAGACCTAAACCACGACC), RNAIII (F- ATTTGTTCACTGTGTCGATAATCC, R- GGAGTGATTTCAATGGCACAAG), and *hla* 7(F- CGAAAGGTACCATTGCTGGTCAGT, R - AAATGCTGAAGGCCAGGCTAAACC). The control primers were for the 16s rRNA gene, as described elsewhere (Koprivnjak et al. 2006).

### Whole human blood survival assay

Survival in whole human blood was performed as previously described (Kolar et al. 2011). In each case, approximately 1 × 10^6^ cfu/mL of exponentially growing cells were inoculated for each strain. These experiments were performed with three separate, deidentified blood samples (purchased from Bioreclamation) and represent nine independent biological replicates. Data are presented as percentage survival of each strain compared with initial inocula. Statistical significance was evaluated using a Student's *t*-test.

### Rabbit blood assay for α-hemolysin activity

Detection of α-hemolysin activity was performed using rabbit blood (Lampire Biological Laboratories, Pipersville, PA), as described previously (Lindsay and Foster 1999).

### Flow cytometry analysis

Phagocytosis of the wild-type and protease-null mutant was analyzed using pooled, whole human blood coupled with flow cytometry. Briefly, a constitutively expressing GFP plasmid (pOS1sGFP::PsarA, Dr Victor J. Torres, gift) was transduced into both strains using phage Φ11, and confirmed by PCR analysis. Strains were then used to infect human blood as described above, before being incubated at 37°C for 4h. After this time, aliquots were removed and APC anti-CD4 and PerCP anti-CD45 antibodies (Becton Dickinson, Franklin Lakes, NJ) were added. These were incubated on ice for 10 min to allow interaction, before erythrocytes were lysed by the addition of RBC buffer (155 mmol/L NH_4_Cl, 100 mmol/L NaHCO_3_, 100 *μ*mol/L EDTA) for 10 min at room temperature. White blood cells were separated by centrifugation at 500 × *g* for 5 min, and cells fixed using 1% formaldehyde solution in 1 × PBS. Cells were then stained with DAPI to ensure that only nuclei-bearing cells were analyzed, and not residual, lysed, RBCs. Cells were then separated according to size, granulocity, antibody staining, and GFP fluorescence using forward and side scattering in an FACs Excalibur cytometer (BD Biosciences, Franklin Lakes, NJ). FACs Diva version 6.1.3 software was used to gate cells and analyze the subsequent data produced. Results represent an average of three independent biological replicates, and are presented as number of GFP-positive cells ± SEM.

### Antimicrobial peptide-sensitivity assay

Liquid cultures of LAC and its protease-null mutant were grown in Luria–Bertani (LB) media without NaCl. These were diluted 1:1000 in fresh LB, again lacking NaCl, and 200 *μ*L was applied to the wells of a 96-well plate. In each case, approximately 1 × 10^6^ cfu/mL of exponentially growing cells were inoculated for each strain. The antimicrobial peptides (AMPs) LL-37, Indolicidin or Histatin-5 were added to these wells in decreasing concentrations and mixed by pipetting. Plates were incubated at 37°C overnight, followed by the measurement of culture density by OD_600_ readings. Data are presented as percentage of inoculum, which was determined by comparing overnight OD_600_ values to no drug controls for each strain. All experiments represent at least three independent biological replicates. Statistical significance was evaluated using a Student's *t*-test.

### Murine model of skin abscess formation

All animal studies in this work were performed in accordance with and approved by, the Institutional Animal Care and Use Committee of the University of South Florida (Permit Number: A-4100-01). For the skin abscess model, experiments were conducted as described previously (Bunce et al. 1992; Shaw et al. 2004). Briefly, 6-week-old, female SKH1-E nude mice were purchased from Charles River Laboratories, and housed at the vivarium in the College of Medicine, University of South Florida. *S. aureus* strains LAC and LAC-protease null were grown for 15 h in TSB as described above. After this time, aliquots of these bacterial suspensions were stored at −80°C, and their cfu/mL determined retroactively by serial dilution and viable cell counts. For infection purposes, cultures were thawed, washed twice in PBS, and diluted in PBS containing 20 *μ*g of sterile Cytodex microcarrier beads to 5 × 10^8^ cfu/mL. Ten mice per strain were inoculated subcutaneously between the scapula with 200 *μ*l bacterial suspension, giving a final inocula of 1 × 10^8^ cfu/mL. Mice were monitored for 6 days during the infectious process, before being sacrificed, and any abscesses harvested and stored at −80°C. Each abscess was subsequently homogenized in 3 mL sterile PBS, and the cfu/abscess determined via serial dilution and viable count enumeration. Statistical significance was evaluated using a Student's *t*-test.

### Murine model of sepsis and dissemination

These experiments were conducted as described previously (Voyich et al. 2006; Li et al. 2009). Briefly, 6-week-old, female CD-1 Swiss mice were purchased from Charles River Laboratories, and housed at the vivarium in the College of Medicine, University of South Florida. *S. aureus* strains LAC and LAC-protease null were prepared as for the skin abscess model. For infection purposes, cultures were thawed, washed twice in PBS, and diluted in PBS to 1 × 10^9^ cfu/mL. Thirty mice per strain were inoculated by tail vein injection with 100 *μ*L bacterial suspension, giving a final inocula of 1 × 10^8^ cfu/mL. The infection was allowed to proceed for 6 days, or until mice reached a premoribund state (used as a measure of mortality). Mice were then euthanized and the brain, liver, kidneys, heart, lungs, and spleens collected and stored at −80°C. Any mouse sacrificed before day 6 was recorded for mortality, but their organs were not analyzed for bacterial burden. Each organ was subsequently homogenized in 3 mL sterile PBS, and the cfu/organ determined via serial dilution and viable count enumeration. The statistical significance of bacterial recovery was evaluated using a Mann–Whitney Test; mortality was measured using a log rank and chi-squared test with 1-degree of freedom.

### Proteomic analysis of surface and secreted proteins

Stationary phase (15 h) cultures of wild-type and mutant strains were prepared in TSB, and their secretomes were harvested and purified as described previously (Rivera et al. 2012). Surface proteins were extracted by methods previously described (Gatlin et al. 2006). Briefly, wild-type and LAC-protease-null mutant cells were grown to stationary phase (15 h) and sedimented via centrifugation. Pellets were resuspended in TSM buffer (100 mmol/L Tris-HCl, 500 mmol/L sucrose, 10 mmol/L MgCl_2_) and incubated in the presence of 100 *μ*g of lysostaphin for 60 min at 37°C. Supernatants were collected and precipitated with 10% trichloroacetic acid, followed by centrifugation to recover precipitates. These were then washed thrice with 100% ice-cold ethanol, before being air-dried. Triplicate samples of secreted and surface proteins for each strain were resuspended in urea buffer, with 15 *μ*g/mL loaded and run on 12% SDS-PAGE gels. At least two lanes were left between each sample to prevent loading contamination, with wild-type and mutant strains run on separate gels. Following this, secreted protein gels were cut into 11 approximately equal fractions, while surface proteins gels were separated into 10 approximately equal fractions. These were then washed with ACN to remove SDS and bromophenol blue, before being dried using a SpeedVac centrifuge (Labconco, Kansas City, MO). Gel pieces were rehydrated with 100 *μ*L of 45 mmol/L DTT and incubated at 55°C for 30 min. The supernatant was removed and replaced with 100 mmol/L iodoacetamide and incubated in the dark at room temperature for 30 min. Following this, the buffer was removed and washed thrice with 50% ACN/50 mmol/L ABC with agitation for 15 min. Gel pieces were dried again using a SpeedVac centrifuge. Promega Trypsin (12.5 ng/*μ*L) was dissolved in Promega trypsin buffer, and enough trypsin solution was used to cover the gel pieces, before incubation at 37°C for 12–16 h. The supernatant was removed and retained, and the reaction stopped with 5% glacial acetic acid. The gel pieces were covered with 100 *μ*L of 50:50 ACN:water containing 0.1% formic acid and vortexed for 15 min. Supernatants were again removed and added to the previously collected supernatants. Samples were dried using a SpeedVac centrifuge, and resuspended in 1 mL of 0.1% formic acid in water. Samples were then desalted and analyzed using a linear ion trap-LTQ instrument mass spectrometer (LTQ XL, Thermo, Waltham, MA) operated using Xcalibur (v2.0.7, Thermo Fisher Scientific, Waltham, MA) data acquisition software, as described by us previously (Rivera et al. 2012).

### Western blotting

Secretome and surfactome samples were prepared as described for the proteomics studies. Samples were standardized to equal protein concentrations and probed using antibodies specific for PVL-LukS (IBT Bioservices, Gaithersburg, MD), Spa (Mark Smeltzer, gift), and SasG (AB Chem, Dorval, Quebec, Canada), as described previously (Shaw et al. 2004). Images are representative from more than three independent biological replicates.

### Statistical analyses

All statistical analyses in this study were performed using SAS software (version 9.2; SAS Institute, Cary, NC). The distribution of data was determined in SAS through tests for normality (*SAS proc univariate*) and equality of variance (*SAS proc ttest*). For parametrically distributed data, a Student's *t*-test was used. The statistical significance of bacterial recovery from the murine model of sepsis was evaluated using a Mann–Whitney test. Mortality was measured using a log rank and chi-squared test with 1-degree of freedom. For all statistical analyses, the significance level was set at α = 0.05.

## Results

### Verification of extracellular protease depletion in the LAC-protease-null strain

An extracellular protease-null strain of *S. aureus* has previously been generated and described in USA300 LAC (Wormann et al. 2011). As it forms the basis of the work described herein, we first set out to confirm the lack of secreted proteases in this strain using a targeted proteomics method. Targeted mass spectrometry uses the inclusion of mass-to-charge ratios in order to guide MS sequencing to a predetermined subset of peptides, and is more accurate than other methods that overlook certain proteins. The Aur, SspA, SspB, ScpA, and SplA (used as a representative for the *spl* operon) sequences were analyzed using the Protein Prospector-MS digest program to determine peptides that would result after trypsin digest. A peptide that is unique to each protease was chosen, and its mass-to-charge ratio included in a designed MS method. Overnight secretome samples (15 h) from the LAC wild type and LAC-protease-null mutant were prepared and analyzed using an LTQ-MS. Spectral counts of proteins identified were compared with the LAC wild type producing robust counts for SspA (48), ScpA (49), SspB (50), Aur (47), and SplA (8). As expected, when the LAC-protease-null mutant secretome was probed, no extracellular proteases were detected. This finding was also verified via gelatin zymogram, with the LAC wild type producing several activity bands, whereas the mutant displayed none (Fig. S1). To ensure that no additional, unintended mutations (specifically *agr*) had occurred in the LAC-protease-null strain, we also performed real-time PCR analysis with primers specific to RNAII, RNAIII, and *hla*, with the latter serving as a representative *agr-*regulated virulence determinant. We found that, at both 5 h and 15 h of growth, there was no observable different between the two strains in the levels of these transcripts (Fig. S2). As such, these analyses verify that an exoprotease-null strain of LAC was created that seemingly bears no apparent additional mutations that impact the synthesis of virulence determinants.

### The protease-null strain has fitness defects during growth in peptide media and serum

**Figure 1:**
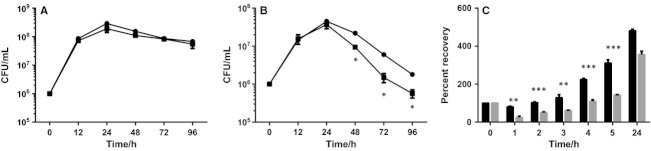
Secreted proteases aid in survival during growth in peptide-based media and serum. The average cfu/mL of the LAC wild type (•) and LAC-protease-null mutant (▪) were compared over 4 days in: (A) TSB and (B) 10% milk broth. (C) The average cfu/mL of the LAC wild type (black) and LAC-ESPN mutant (gray) were compared at the times indicated during growth in pig serum. Data are expressed as percent recovery of the inoculum. Presented is the average data from three independent biological replicates. In all cases, a Student's *t*-test was used to determine statistical significance, *P = 0.05, **P = 0.01, ***P = 0.005. Error bars are shown ±SEM.

We first sought to assess whether loss of extracellular proteases affected viability of the USA300 LAC strain. As such, growth profiling was performed in complex media (TSB) over a 96-h period, with no difference observed compared to the parental strain (Fig. 1A). Additionally, when using chemically defined media containing only glucose and free amino acids as nutritive sources, we also saw no alterations in growth between the strains (data not shown). These findings are perhaps to be expected, as, for example, *agr* mutants of *S. aureus* are aproteolytic, yet are undoubtedly viable in complex media. Following this, we next tested the ability of the protease-null strain to grow in peptide-based media. This was performed as many bacterial species possess a PrtP homolog, which is a surface-exposed protease that functions to generate oligopeptides from polypeptides for nutrition (Siezen 1999; Liu et al. 2010). Such a protein is lacking in *S. aureus* (our unpublished observation), and therefore we reasoned that the extracellular proteases perhaps fulfill such a function. Accordingly, we grew the LAC parental strain and its protease-null mutant in 10% milk broth (Fig. 1B), which is routinely used to evaluate peptide-based nutrition (Borezee-Durant et al. 2009), as it contains few free amino acids, and abundant peptides. During the first 24 h of profiling, no difference in viability for the LAC wild-type and its protease-mutant strain was observed. Interestingly, after this time, both strains began to lose viability, with the protease-null strain proving significantly less able to resist starvation compared with the parent. Specifically, by day 2, the protease mutant produced a 2.3-fold decrease in average cfu/mL, which was exacerbated at day 3 with a fourfold decrease in survival. By day 4, a 3.1-fold decrease in mutant viability was determined.

We next set out to assess the fitness of the wild type and protease mutant during growth in a more pathogenically relevant medium, in this case pig serum. Pig serum was chosen due to ease of availability, and because it effects have been shown previously to closely mimic those of human serum (Wiltshire and Foster 2001). Accordingly, exponentially growing cells of the parent and mutant were inoculated into pig serum, and the survivability of three biological replicate samples determined (Fig. 1C). When growth was analyzed during early time points, a large decrease in the viability of the protease null strain was observed. Specifically, after 1 h, a 3.2-fold decrease in mutant cell viability was observed (24.6% of the inoculum), compared with the parent strain (80.9% of the inoculum). Viability stabilized after this time, yet still produced a consistent twofold reduction in the mutant strain compared to the parent from 2 to 5 h. After 24 h of incubation, while both strains had robust growth (Wt = 480.8% recovery of inoculum, protease-null strain = 355.6%), there was still a decrease in final loads of the mutant strain. This suggests that secreted proteases may have a role in nutrition acquisition in *S. aureus*, particularly during times of stress and starvation.

### Secreted proteases play a role in resistance to antimicrobial peptides

**Figure 2:**
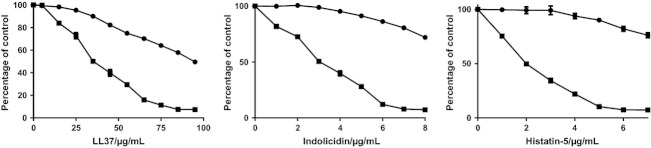
Secreted proteases play a role in the resistance of *S. aureus* to antimicrobial peptides. The LAC wild type (•) and protease-null mutant (▪) were cultured with either LL-37, indolicidin or histatin-5. Values represent percentage of overnight OD_600_ readings for each concentration compared with a no-drug control for each strain. Data presented is from at least three independent biological replicates. In all cases, a Student's *t*-test was used to determine statistical significance, with every point other than the first LL-37 concentration proving to be a minimum of *P* = 0.05, with most being in excess of *P* = 0.001. Error bars are shown ±SEM.

The specific mechanism by which the reduced viability of the protease-null strain in serum is mediated is unclear. Serum contains a number of antibacterial components that may contribute to this growth defect, including complement. However, as there are no cell-mediated components of the immune system within serum, complement would not kill *S. aureus* cells in these assays. More likely, the decreased viability of the mutant strain is explained by the fact that serum also contains abundant AMPs. In support of this, it has previously been shown that the *S. aureus-*secreted protease, aureolysin, is able to cleave the human cathelicidin, LL-37 (Sieprawska-Lupa et al. 2004). To determine if secreted proteases facilitate resistance to only this AMP, or other such peptides, sensitivity profiling of the mutant was performed (Fig. 2). While both strains exhibited declining viability at increasing concentrations of the AMP LL-37 (compared with their no-drug controls), the protease-null strain was considerably more impaired. Specifically, we observed a twofold decrease in protease-null cells compared with the parental strain at a concentration of 45 *μ*g/mL, which increased to >fourfold at 65 *μ*g/mL, and more than 7.5-fold at 85 *μ*g/mL. Similar findings were observed with indolicidin, with a 2.5-fold decrease in viability observed for the mutant strain at 4 *μ*g/mL, a more than sevenfold observed at 6 *μ*g/mL, and >10-fold at 7 *μ*g/mL. Finally, when these analyses were performed using histatin-5 we observed a >2.5-fold decrease in mutant viability at 3 *μ*g/mL, a >8.5-fold at 5 *μ*g/mL, and >11-fold at 6 *μ*g/mL. Collectively, it is apparent that extracellular protease deletion results in heightened sensitivity of *S. aureus* cells to the effects of antimicrobial peptides; and that this observation is not limited to a single peptide example. As such, these findings strongly suggest that secreted proteases are a major mechanism by which *S. aureus* resists the toxic effects of AMPs.

### Extracellular proteases are required for survival during interaction with the innate immune system

**Figure 3:**
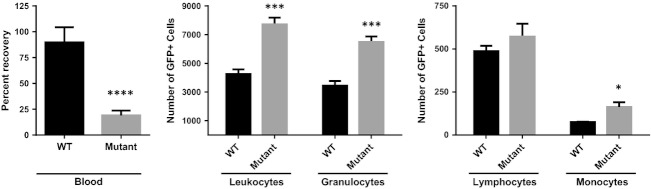
Secreted proteases are protective during interaction with the human innate immune system. The LAC wild-type and protease-null strain were separately cultured in whole human blood for 4 h, before their viability was determined. Data is expressed as percent recovery of the inoculum, and represents three separate blood samples and nine individual biological replicates. The phagocytosis of these strains in whole human blood was also measured by FACs analysis using GFP-labeled variants grown in an identical manner. Data are expressed as number of GFP+ cells for the mutant and wild type in the various types of white blood cell. Numbers are derived from three biological replicates. Error bars are shown ±SEM. Statistical significance was calculated using a Student's *t*-test, *P = 0.05, ***P = 0.005, ****P = 0.001.

The in vitro AMP sensitivity assays suggest that extracellular proteases are important for survival during interaction with elements of the immune system. Accordingly, we next profiled survivability of the mutant strain in the presence of the innate immune system, including its cellular components. As such, exponentially growing LAC wild-type and protease-null mutant cells were separately cultured in whole human blood for 4 h, and their viability determined (Fig. 3). Analysis revealed that the LAC wild type had bacterial loads that decreased only marginally from the initial inoculum (82.1% recovered). In contrast, the protease-null mutant displayed significantly impaired survival, returning only 12.6% of the inoculum. This results in a 6.5-fold reduction in survivability of the mutant strain when compared with the parent. To understand the reason for this decline, we next analyzed phagocytosis of the two strains during infection in whole human blood using flow cytometry. This was achieved using variants of these two strains bearing a plasmid that constitutively expresses *gfp*. These strains were incubated under identical conditions to the whole human blood survival assay, before white blood cells were isolated and subjected to separation and analysis by FACS (Fig. 3). Importantly, when looking at all leukocytes collectively, we observed an approximate twofold increase in the number of protease-null mutant cells that had been phagocytosed, when compared with the parental strain. When performing more detailed analyses for specific cell types, we again saw an equivalent twofold increase in protease-null cells within both granulocytes and monocytes. Interestingly, when analyzing lymphocytes, we did not see a meaningful or statistically significant fold change for the mutant compared to the parental strain. Ultimately, these data strongly suggest that secreted proteases play a key role in protecting *S. aureus* during interaction with the human immune system.

### Secreted proteases contribute to CA-MRSA skin abscess formation

**Figure 4:**
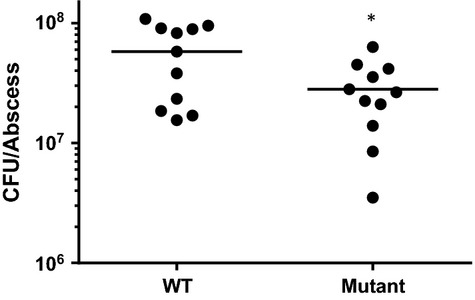
Secreted proteases contribute to CA-MRSA skin abscess formation. The LAC wild type and protease-null mutant were used to subcutaneously inoculated the scapula of 10 SKH-1 mice each, at 1 × 10^8^ cells. After 6 days, abscesses were harvested and bacterial loads determined via homogenization and serial dilution. The average cfu/abscess returned is indicated by horizontal bars. A Student's *t*-test was used to determine statistical significance, *P = 0.05.

There are conflicting results regarding the role of extracellular proteases in *S. aureus* virulence (Coulter et al. 1998; Reed et al. 2001; Rice et al. 2001; Calander et al. 2004; Shaw et al. 2004; Imamura et al. 2005). As such, we set out to determine if the 10 major secreted proteases collectively contribute to localized infection using a murine model of skin abscess. Accordingly, 10 mice were subcutaneously inoculated with either the LAC wild type or protease-null mutant, and the infection was allowed to proceed for 6 days. Following this, all mice were euthanized, any abscesses harvested, and the bacterial loads per abscess determined (Fig. 4). We observed that the protease null mutant had significantly reduced bacterial loads per abscess when compared with the wild type. Specifically, the average wild-type cfu/abscess was 57.7% of the inoculum, whereas for the mutant it was 28.1%, representing a twofold decrease in bacterial burden.

### Extracellular proteases play a key role during systemic CA-MRSA infections

**Figure 5:**
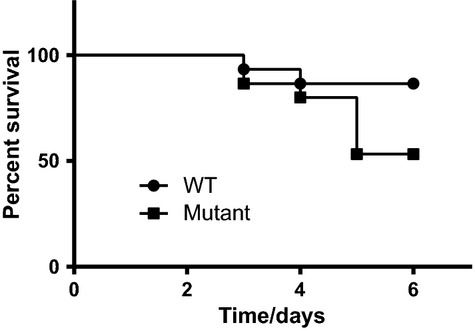
Deletion of secreted proteases results in increased mortality during systemic CA-MRSA infection. The LAC wild type and protease mutant were inoculated via tail vein injection into 30 CD-1 mice each, at 1 × 10^8^ cells. The infection was allowed to proceed for 6 days, or until mice reached a premoribund state (used as a measure of mortality). A log rank and chi-squared test with 1-degree of freedom were used to determine statistical significance (*P* = 0.067).

In addition to presenting as skin and soft tissue infections, CA-MRSA is also a major cause of bacteremia and systemic disease. As such, we next set out to determine if extracellular protease deletion impacts systemic CA-MRSA infections. Accordingly, mice were inoculated via tail vein injection with 1 × 10^8^ cells of either the LAC wild type or protease-null mutant. The infection was allowed to proceed for 6 days, or until mice reached a premoribund state (used as a measure of mortality). Mice were then euthanized and the brain, liver, kidneys, heart, lungs, and spleens collected. Any mouse sacrificed before day 6 was recorded for mortality, but their organs were not analyzed for bacterial burden. Following recovery, each organ was homogenized, serial diluted, and plated to determine bacterial load. Of the 30 LAC wild-type animals, two died prior to day 6, yielding a mortality rate of 6.6% (Fig. 5). In contrast, seven of the 30 inoculated with the LAC-protease null mutant died, giving a 23.3% mortality rate. This represents a 3.5-fold increase in mortality for the protease-null strain compared with the parental strain. Statistical analysis of this finding resulted in a *P-*value = 0.067, which is just outside the range of statistical significance.

**Figure 6:**
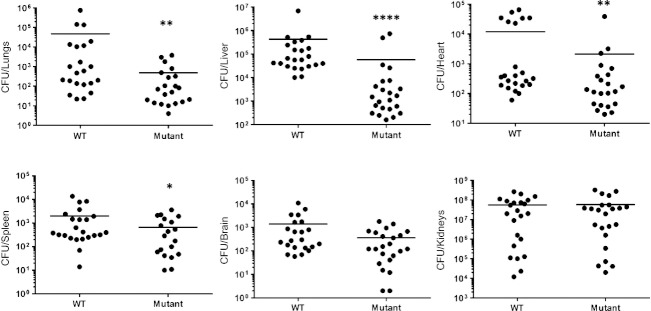
Secreted proteases are required for systemic dissemination during septic CA-MRSA infections. The LAC wild type and protease mutant were inoculated via tail vein injection into 30 mice each, at 1 × 10^8^ cells. After 6 days, all surviving mice were euthanized, and their organs harvested for bacterial load derivation. A Mann–Whitney test was used to determine statistical significance. *P = 0.05, **P = 0.01, ****P = 0.001.

Conversely, when analyzing bacterial burden per organ between the two groups, we observed significant decreases for the mutant strain in the liver, lungs, heart, and spleen (Fig. 6). Specifically, the largest fold change, 98.7, was observed in the lungs, with the LAC wild type returning an average cfu/lungs of 4.74 × 10^4^ and the protease null mutant returning 4.80 × 10^2^. Additionally, we observed a 7.4-fold decrease in bacterial load in the liver of inoculated mice, with the wild type producing an average cfu/liver of 4.32 × 10^5^ and the mutant 5.79 × 10^4^. The heart produced a 5.7-fold decrease in mutant bacterial cells, with LAC yielding 1.2 × 10^4^ and the protease mutant yielding 2.1 × 10^3^. With regards to the spleen, we observed 2.01 × 10^3^ cfu/spleen for the parental strain and 6.6 × 10^2^ for the mutant, which is a threefold decrease. Finally, in the brain we observed a 3.8-fold decrease in the average cfu/brain for mutant-infected mice, however, this was found to be outside the range of significance (*P* = 0.079). Interestingly, there was no significant difference between the two strains in their ability to infect the kidneys of inoculated mice. The LAC wild type returned 5.57 × 10^7^ cfu/organ, whereas the protease-null mutant returned 5.87 × 10^7^. Collectively, these data suggest that secreted proteases play a major role in the survival of *S. aureus* during in vivo infection.

### Secreted proteases are primary effectors of virulence-determinant stability

It has previously been proposed (McGavin et al. 1997; Lindsay and Foster 1999; Karlsson et al. 2001; McAleese et al. 2001; Zielinska et al. 2011; Gonzalez et al. 2012) that secreted proteases regulate the stability of self-derived toxins. To determine if this is in fact the case, we assessed the impact of secreted proteases on virulence-determinant stability using proteomic techniques. Accordingly, stationary-phase secretomes (15 h) of the LAC wild type and protease-null mutant were collected in triplicate and separated via SDS-PAGE. Each gel was then cut into 11 identical fractions to facilitate processing. The data from all 11 fractions were pooled and collectively analyzed for alterations in protein abundance using spectral counts (Table S1). In total, 19 known secreted proteins had increased abundance of 1.5-fold or greater upon deletion of extracellular proteases. The highest fold change was seen for phenol-soluble modulin alpha 4, with a 4.2-fold increase in the mutant. Other well-known virulence determinants were also found to be more abundant in the protease-null strain, including two Lipases (Geh = 3.8-fold, SAUSA300_2603 = 2.5-fold), components of the γ-hemolysin (HlgA = 2.4-fold, HlgC = 2.1-fold), α-toxin (twofold), leukotoxin LukE (twofold), and enterotoxin Q (1.5-fold). Our analysis also revealed increased abundance of certain proteins in the wild-type strain, including all 10 secreted proteases, as expected, along with catalase (ninefold), the CamS pheromone (twofold), and two putative lipoproteins (SAUSA300_2403 = twofold, SAUSA300_0411 = threefold).

**Figure 7:**
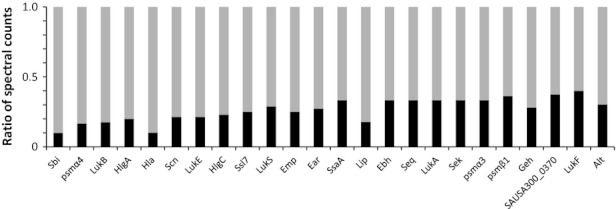
Extracellular proteases modulate the stability of a wealth of known secreted virulence factors**.** The secretome of LAC and its protease-null strain were collected and fractionated via SDS-PAGE. Known, full-length secreted proteins were analyzed and their spectral counts determined. Shown are the ratios of abundance for each secreted protein in the wild type (black) compared to the mutant (gray).

A consideration with this pooled analysis is that a protein may be processed by secreted proteases into potentially inactive fragments, as a result of endoproteolysis. As the protein is not completely degraded to free amino acids, each of these fragments will be detected by MS analysis; therefore one may not observe any fold change in spectral counts, yet cleavage has rendered the protein nonfunctional. As such, protein abundance was next compared within individual fractions from the SDS-PAGE gels. Spectral counts for a given protein were only analyzed for the single fraction that would contain its full-length protein, based on predicted molecular weight. (Fig. 7 and Table S2). The 19 proteins identified from the pooled sample all had decreased abundance in the wild-type strain in their relative molecular weight fractions; in many cases to even greater degrees than in our collective analysis. For example, we observed increased abundance for Sbi (ninefold), alpha-toxin (8.8-fold), phenol-soluble modulin alpha 4 (fivefold), γ-hemolysin (HlgA = fourfold), and LukE (3.6-fold) upon deletion of extracellular proteases using this method. Furthermore, an additional five proteins that did not produce a significant fold change in the pooled samples were identified as being altered in protein stability in the mutant strain. These include the two components of the Panton-Valentine leukocidin (LukS = 2.4-fold, LukF = 1.5-fold), the staphylococcal complement inhibitor (3.6-fold), enterotoxin K (twofold), and secretory antigen SsaA (twofold).

**Figure 8:**
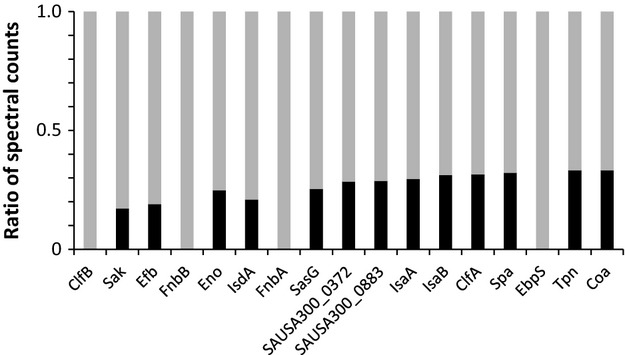
Extracellular proteases modulate the stability of a number of surface-associated virulence factors. The surfactome of LAC and its protease-null strain were collected and fractionated via SDS-PAGE. Known, full-length surface proteins were analyzed and their spectral counts determined. Shown are the ratios of abundance for each surface protein in the wild type (black) compared to the mutant (gray).

Following this, we also performed a similar analysis for surface proteins between the two strains. The rationale for this is that existing evidence in the literature suggests secreted proteases can also target surface-exposed proteins in *S. aureus* (McGavin et al. 1997; Karlsson et al. 2001; McAleese et al. 2001). As such, the cells used to generate secretomes were harvested, and their surface protein fraction isolated, before being processed in an identical SDS-PAGE-based fashion. When performing a collective analysis of spectral counts for all fractions, we observed 10 proteins with a 1.5-fold or greater increase in the protease-null mutant (Table S3). These include fibronectin-binding proteins A (fourfold) and B (threefold), as well as fibrinogen-binding protein (2.3-fold), clumping factor A (1.7-fold), and IsdA (1.6-fold). We also performed specific fraction analysis as detailed above to determine those proteins with altered protein stability upon deletion of the extracellular proteases (Fig. 8 and Table S4). Specific analysis revealed that, in addition to the 10 surface proteins identified from pooled studies, a further seven had increased stability in the protease mutant. These included immunodominant staphylococcal antigens A (2.3-fold) and B (2.2-fold), staphylocoagulase (twofold), immunoglobulin G-binding protein A (Spa = 2.1-fold), elastin-binding protein (twofold), and a putative surface protein (SAUSA300_0883 = 2.4-fold). We again observed a general increase in protein abundance for the majority of proteins identified by fraction analysis when compared with the pooled data. The most striking of which was clumping factor B, which, upon fraction analysis was found at levels eightfold higher in the mutant.

In order to independently corroborate our findings from these proteomics studies, we performed Western blot analysis for selected secreted and surface-associated virulence factors. Identical 15-h secretome and surfactome samples were prepared as described for the proteomics studies, and separately probed for PVL-LukS, SasG, or Spa. In each case (Fig. S3), we observed decreased accumulation of these proteins in the parental strain when compared to the protease-null mutant. Furthermore, to demonstrate that protease inactivation not only leads to increase protein stability, but also biological activity, we profiled the cytolytic activity of Hla toward rabbit erythrocytes. As expected, we observed a significant increase in α-hemolysin activity in the protease-null strain compared with the wild type (Fig. S4). These findings support our proteomic analyses, and are consistent with nonproteomic findings from our previous works, showing increased stability of α-toxin, and the psms, upon protease disruption (Zielinska et al. 2011).

## Discussion

Recent reports suggest that MRSA is now the leading cause of infectious disease and death by a single agent in the U.S. (Camargo and Gilmore 2008; Kobayashi and DeLeo 2009). This pathogenic success can largely be attributed to the meteoric increase in CA-MRSA infections in the last decade (Moran et al. 2006; Johnson et al. 2007; Popovich et al. 2008; Webb et al. 2009). Although several CA-MRSA lineages exist, USA300 is now thought to account for more than 50% of all MRSA infections in some regions (Thurlow et al. 2012). The reason for the surprising success of USA300 is somewhat unclear; however, work by a number of groups suggests it may be attributable to the differential expression of core genomic elements (Li et al. 2009), including the PSMs, hemolysins, extracellular proteases (Fig. S5), and enterotoxins (Wang et al. 2007; Diep and Otto 2008; Montgomery et al. 2008; Li et al. 2009). A number of studies performed by ourselves and others have investigated the contribution of extracellular proteases to disease causation (Coulter et al. 1998; Reed et al. 2001; Rice et al. 2001; Karlsson and Arvidson 2002; Sifri et al. 2003; Calander et al. 2004; Shaw et al. 2004; Imamura et al. 2005; Kubica et al. 2008; Prajsnar et al. 2008). Thus far, these data have proved contradictory, perhaps as a result of using strains that have varying proteolytic capabilities. Some backgrounds used (e.g. RN6390) have limited clinical significance, while other studies have focused only on individual protease mutations. This latter point is of particular importance as it has been shown in other highly proteolytic bacteria that significant levels of functional redundancy exist (Travis et al. 1995). For these reasons, and to explore the role of this class of enzymes in CA-MRSA pathogenesis, we have focused in this study on a total exoprotease-null mutant of USA300 LAC.

Interestingly, while we show that the mutant strain is viable in complex media, we did observe a survival defect during extended culturing in peptide-based media. Previous reports have shown that *S. aureus* cells can only import octapeptides or smaller for nutrition-based purposes (Hiron et al. 2007). Therefore, it is possible that the extracellular proteases of *S. aureus* cleave large oligopeptides into smaller fragments for growth and nutrition. Interestingly, the defect was only apparent after 24 h of growth. Thus, it is possible that at earlier time points, the major nutrient source is carbon, free amino acids, and oligopeptides with less than eight residues. Once these more readily usable nutrients have been exhausted, the focus would then shift to breaking down oligopeptides with greater than nine residues. In support of this assertion, others (Borezee-Durant et al. 2009) have suggested that *S. aureus* grown in milk during earlier time points uses simple energy sources such as sugars and free amino acids, before switching to more complex oligopeptides during later growth phases. In this scenario, any strain lacking extracellular proteases would be limited in their ability to generate smaller, importable peptides for nutrition during later growth phases, explaining the survival defect observed. This contention is supported by the observation that, unlike a variety of other organisms, *S. aureus* lacks a PrtP homolog, which serves to breakdown large oligopeptides for import and nutrition (Siezen 1999; Liu et al. 2010). We also observed fitness defects in the protease-null strain during growth in the more pathogenically relevant pig serum, whose effects have previously been shown to closely mimic those of human serum (Wiltshire and Foster 2001). These findings are perhaps a result of an inability to degrade proteins for nutritive purposes. In support of this, others have shown that nutrition defects become emphasized during growth in serum (Beasley et al. 2009). Additionally, it has also been shown that extracellular proteases are strongly upregulated during the growth of *S. aureus* in serum (Oogai et al. 2011); further suggesting that they may be important for survival under these conditions. Serum also contains complement, which is a key mediator of the immune response to bacterial infection within the host. However, as there are no cell-mediated components of the immune system within serum, complement would have no ability to kill *S. aureus* cells in our assays. Therefore, despite reports detailing the role of secreted proteases in complement evasion (Laarman et al. 2011); this would seemingly have little to do with the survival defect observed. More likely, the decreased viability of the mutant strain is explained by the fact that serum contains abundant AMPs.

This contention is corroborated by our observation that the protease mutant strain is more sensitive to AMPs. It has previously been shown that aureolysin cleaves the cathelicidin AMP, LL-37 (Sieprawska-Lupa et al. 2004). Herein, we show that extracellular proteases facilitate resistance to agents other than LL-37, as we observed increased sensitivity to both indolicidin and histatin-5. During infection, AMPs bind to bacterial cells through cationic interactions at the cell surface, and exhibit antibacterial activity through several mechanisms (Brogden 2005). Therefore, in *S. aureus*, one would hypothesize that secreted proteases aid in immune evasion by preemptively degrading these agents. We further investigated this assertion by evaluating survival in whole human blood. In addition to complement and AMPs, whole human blood also contains cellular components of immunity. When such profiling was performed we also saw major survival defects in the mutant strain. The immune components of serum and blood are relatively similar, and thus the decreases in survivability observed may result from a lack of AMPs and complement system degradation in the mutant strain. However, in serum at 4 h, the mutant produced a twofold decrease, while in blood, a 6.5-fold decrease was observed at the same time.

To further understand these findings, we performed FACS analyses using GFP-labeled variants of the wild-type and protease-null strain grown in human blood. After 4 h of incubation we observed increased phagocytosis of protease-null cells compared with the parental strain. This is in keeping with work by others, which shows that Staphopain B can cleave both CD11b and CD31, which block the phagocytosis of *S. aureus* by neutrophils and monocytes (Smagur et al. 2009a,b). Furthermore, recent work has shown that Staphopain A also contributes to immune evasion by cleaving the chemokine receptor CXCR2 (Laarman et al. 2012). Additionally, aureolysin is known to inhibit phagocytosis of *S. aureus* by neutrophils via the cleavage of C3 (Laarman et al. 2011). Our group has also shown that a functional aureolysin is required to survive phagocytosis by human macrophages (Kubica et al. 2008). Finally, a number of the secreted proteases have been shown to cleave components of human blood, such as prothrombin and pro-uPA, in order to facilitate survival and proliferation (Wegrzynowicz et al. 1980; Beaufort et al. 2008). Collectively, one hypothesizes that the survival defect of the protease-null strain in human blood results from a reduced capacity to evade the action of AMPs, alongside increased phagocytosis by leukocytes, ultimately leading to enhanced killing within the phagosome. Collectively, findings from the literature, along with our data presented herein, strongly argue that extracellular proteases play a protective and beneficial role during interaction of *S. aureus* with the host immune system.

We also observed significant influence of the secreted proteases on *in vivo* pathogenesis. This was first studied for localized infection, using a murine model of abscess formation. Over the course of a 6-day period, we observed an approximately twofold decrease in virulence for the mutant strain. This decrease is perhaps not as large as would be expected; previous work has shown that 8325-4 *sspA* and *sspB* mutants are more than threefold impaired in virulence when using this same model (Shaw et al. 2004). The reason why a complete protease-null mutant would display less attenuation than single protease mutants is likely explained by the proteomics works conducted in this study. We observe that upon deletion of the secreted proteases, the stability of a large number of key virulence factors increases significantly. As such, a protease-null strain actually accumulates more toxins, rather than less. This is particularly important in this model, as key mediators of skin infections, such as α-toxin and the PSMs, are more abundant upon protease inactivation. Therefore, the observed twofold reduction is actually quite surprising, given the increased prevalence of abscess-enhancing toxins. As such, any observed decrease at all upon protease deletion is a major finding, and suggests that the loss of these enzymes, despite being subverted by other abscess-impacting agents, influences the ability of *S. aureus* to cause localized disease.

When using systemic infection models, we observed further, and profound alterations in virulence that were far in excess of those seen for localized infections. Specifically, the mutant displayed significantly decreased bacterial loads in the lungs, liver, heart, and spleen. The specific explanations for these findings, are likely complex and multifactorial. However, it has previously been shown that secreted proteases can cleave human α1-proteinase inhibitor (Potempa et al. 1986), α1-antichymotrypsin, the heavy chains of all human immunoglobulin classes (Prokesova et al. 1992), elastin (Potempa et al. 1988), fibrinogen, fibronectin, high-molecular-weight kinininigen, and plasminogen (Wegrzynowicz et al. 1980; Potempa et al. 1986, 1988; Massimi et al. 2002; Imamura et al. 2005; Beaufort et al. 2008). Cleavage of each of these host proteins aids in tissue invasion, and perhaps explains the decrease in mutant cells in these organs. Furthermore, we demonstrate in our proteomics studies that protease-null mutant cells have increased decoration of their cell walls, as higher levels of surface proteins were observed upon deletion of proteolytic enzymes. Indeed, our preliminary studies have shown that the protease-null strain is significantly more adhesive to surfaces coated with human proteins, including elastin (our unpublished observations). This coupled with our findings that the protease mutant strain is less resistant to AMPs, and has decreased survival in both serum and whole blood, might begin to explain this phenotype. As such, one would hypothesize that upon inoculation into the blood, the protease-null strain would immediately display decreased fitness for survival, increased attachment, decreased dissemination and invasion, and an inability to cleave key host proteins for survival, evasion, and nutrition.

In stark contrast to this, we in fact observe increased killing by the protease-null mutant when mortality was used as a measure of infection. This finding is of primary importance, and speaks strongly to a major role for the secreted proteases in controlling, and tightly regulating the infectious process. Herein, we show major increases in virulence-determinant stability upon inactivation of extracellular proteases. Specifically, we show that α-toxin, γ-hemolysin, PSMs, LukE, LukAB, PVL, and others are all more abundant in the protease-null strain. Importantly, each of these factors have been linked to increased virulence and mortality during *S. aureus* infection (Morinaga et al. 2003; Bubeck Wardenburg et al. 2007; Labandeira-Rey et al. 2007; Wang et al. 2007; Loffler et al. 2010; Dumont et al. 2011). Furthermore, work by McAdow and coworkers has shown that bacterial agglutination in blood, mediated by ClfA, Coa, and vWbp, is associated with lethal outcomes of *S. aureus* septic infections in mice (McAdow et al. 2011). As we see elevated stability of both ClfA and Coa in the protease-null strain, this may further explain the elevated mortality observed. Thus, despite the loss of extracellular proteases and their effects on the host, other major virulence factors are more prevalent and stable. We propose that in the absence of proteolytic activity, these other virulence factors exist unchecked, and therefore provide the potential for the aggressive progression of infection observed. Indeed, the overproduction of PVL has frequently been linked to the rapid and increased mortality associated with necrotizing pneumonia (Morgan 2005; Garnier et al. 2006). Such a scenario is supported by reports on the role of the cysteine protease, SpeB, from group A Streptococci (GAS). When levels of this enzyme rise in GAS, the stability and abundance of other virulence factors is reduced, leading to impairments in invasion and virulence (Chatellier et al. 2000; Kansal et al. 2000, 2003).

A major consideration with each of our phenotypic and growth-related studies is that the data presented may be skewed by elevated aggregation of the protease-null strain. It has previously been reported that secreted extracellular proteases have been shown to cleave surface and adhesive proteins by other groups (McGavin et al. 1997; Karlsson et al. 2001; McAleese et al. 2001). Therefore it is reasonable to project that the protease-null strain may display elevated clumping compared with the parental strain, as we intimate above. As a large part of our experimental design was dependent on accurate cfu/mL values, we performed prior analyses to ensure that our data was not influenced by such aggregative behavior. We determined that extended vortexing produced identical cfu/mL values to cellular disruption via sonication for both strains in every growth condition and animal model used (data not shown). As such, all cfu/mL values reported herein are the result of phenotypic variations, rather than increased clumping of the protease-null strain.

Therefore, this presents a scenario where, in addition to their own, independent virulence-affecting roles, the secreted proteases exist as key check point enzymes to control the severity and intensity of infection via modulating the stability of other toxins and virulence determinants. Indeed, one could suggest that the regulatory control of secreted proteases by the *agr* system is no accident, but in fact evolutionary design. In such a scenario, any enhancement of *agr* activity, such as that seen in CA-MRSA strains, would lead to massive toxin production, and death of the host organism. As an opportunistic pathogen, such rapid killing of the host by *S. aureus* is counterintuitive to survival. Therefore, by tying the production of other toxins to extracellular protease expression and activity, one is presented with an inbuilt mechanism to self-regulate the overtly harmful and often lethal effects of secreted toxins on the host, thus tempering and controlling the pathogenic process.

In summary, we demonstrate that the extracellular proteases of *S. aureus* play a variety of key roles in the virulence process. Specifically, they aid in protection against the innate immune system, at both cell-dependent and independent levels. They also strongly impact the progression of localized and systemic CA-MRSA infections. Finally, and perhaps most importantly, they are key mediators of secreted and cell wall-associated virulence-determinant stability. Collectively our findings provide a unique insight into the progression of CA-MRSA infections, and the role of secreted proteolytic enzymes.

## Supporting Information

**Figure S1.** Gelatin zymography of the LAC wild-type and its protease-null mutant.

**Figure S2.** Exoprotease deletion does not lead to observable difference in *agr* or *hla* transcript levels.

**Figure S3.** Western blot analysis confirms increased abundance of select surface and secreted virulence factors upon exo-protease deletion.

**Figure S4.** Exoprotease deletion leads to increased α-hemolysin activity.

**Figure S5.** Secreted proteolytic activity of clinical *S. aureus* isolates.

**Table S1.** Collective alterations in protein abundance for secreted proteins upon deletion of extracellular proteases in *S. aureus* USA300.

**Table S2.** Individual fraction analysis of alterations in protein stability for secreted proteins upon deletion of extracellular proteases in *S. aureus* USA300.

**Table S3.** Collective alterations in protein abundance for surface proteins upon deletion of extracellular proteases in *S. aureus* USA300.

**Table S4.** Individual fraction analysis of alterations in protein stability for surface proteins upon deletion of extracellular proteases in *S. aureus* USA300.
