# Supplementary material for: Extracellular proteases are key mediators of Staphylococcus aureus virulence via the global modulation of virulence-determinant stability
Source: Microbiologyopen. 2012 Dec 11;2(1):18–34. doi: 10.1002/mbo3.55 (PMC3584211; doi:10.1002/mbo3.55)
Supplement: Supplementary file 1 [file mbo30002-0018-SD1.pdf]

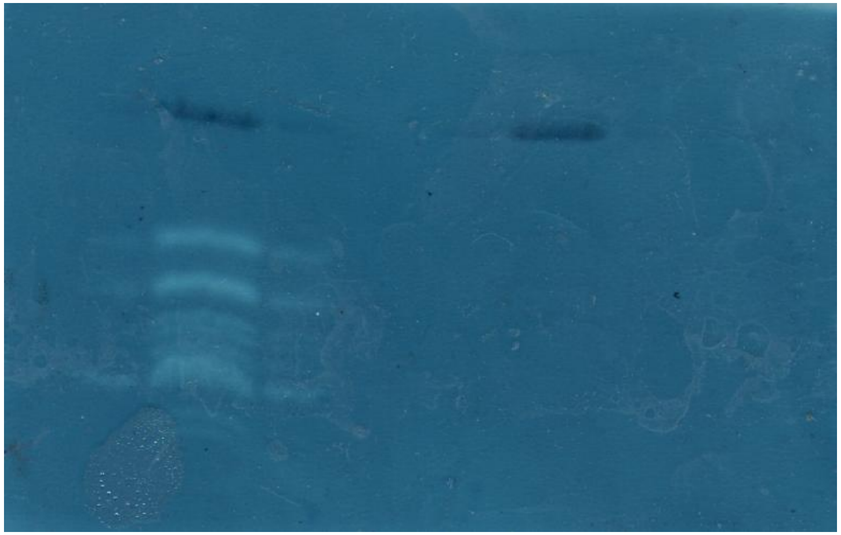

Supplemental Figure 1. Gelatin zymography of the LAC wild-type and its protease-null mutant. The secretomes of the LAC wild-type and protease-null mutant were collected (15h) and protease activity visualized on a zymogram gel containing gelatin, as described by us previously [Shaw et al., 2004].

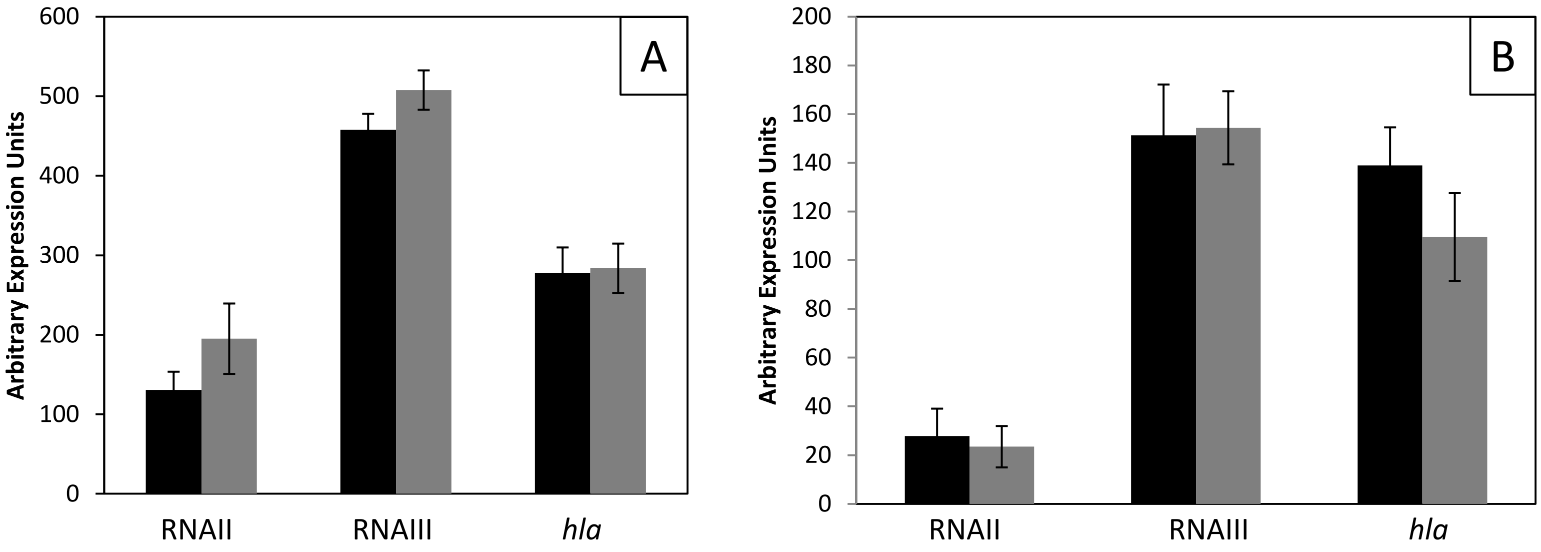

Supplemental Figure 2. Exoprotease deletion does not lead to observable difference in *agr* or *hla* transcript levels. qRT-PCR was performed with primers specific to RNAII, RNAIII and *hla* in the LAC wild-type (black) and protease-null (grey) strains. These analyses were performed at (**A**) 5 hours and (**B**) 15 hours of growth. The data presented are from at least three independent cultures; error bars are shown as ±SEM.

Supplemental Table 1. Collective alterations in protein abundance for secreted proteins upon deletion of extracellular proteases in *S. aureus* USA 300.

Protein	Gene	Accession Number	WT[a,c]	M[b,c]	Fold Change
Phenol-soluble modulin alpha 4 peptide	*psmα4*		4	17	4.25
Triacylglycerol lipase	*geh*	SAUSA300_0320	276	1062	3.84
Autolysin	*atl*	SAUSA300_1921	117	303	2.58
Triacylglycerol lipase	*lip*	SAUSA300_2603	168	425	2.52
Gamma-hemolysin component A	*hlgA*	SAUSA300_2365	29	72	2.48
Gamma-hemolysin component C	*hlgC*	SAUSA300_2366	17	37	2.17
Alpha-hemolysin	*hla*	SAUSA300_1058	226	462	2.04
Staphylococcal superantigen-like protein 7	*ssl7*	SAUSA300_0401	2	6	2
Extracellular matrix-binding protein	*ebh*	SAUSA300_1327	2	4	2
Extracellular matrix protein-binding protein	*emp*	SAUSA300_0774	5	10	2
Leukocidin	*lukA*	SAUSA300_1975	88	176	2
Leukotoxin	*lukE*	SAUSA300_1769	8	16	2
Leukocidin	*lukB*	SAUSA300_1974	83	157	1.89
Putative staphylococcal enterotoxin		SAUSA300_0370	7	13	1.85
Ear protein	*ear*	SAUSA300_0815	8	14	1.75
Phenol-soluble modulin beta 1 peptide	*psmβ1*	SAUSA300_1067	5	8	1.6
Staphylococcal enterotoxin Q	*seq*	SAUSA300_0801	12	19	1.58
Immunoglobulin-binding protein	*sbi*	SAUSA300_2364	17	26	1.52
Phenol-soluble modulin alpha 3 peptide	*psmα3*		2	3	1.5
Putative lipoprotein		SAUSA300_2403	2	0	-2
Sex pheromone	*camS*	SAUSA300_1884	2	0	-2
Uncharacterized lipoprotein		SAUSA300_0411	3	1	-3
Catalase	*katA*	SAUSA300_1232	9	0	-9
Serine protease	*splA*	SAUSA300_1758	14	0	-14
Serine protease	*splD*	SAUSA300_1755	15	0	-15
Zinc metalloproteinase aureolysin	*aur*	SAUSA300_2572	15	0	-15
Serine protease	*splE*	SAUSA300_1754	18	0	-18
Serine protease	*splC*	SAUSA300_1756	18	0	-18
Serine protease	*splF*	SAUSA300_1753	27	0	-27
V8 protease	*sspA*	SAUSA300_0951	43	1	-43
Serine protease	*splB*	SAUSA300_1757	46	0	-46
Cysteine protease	*sspB*	SAUSA300_0950	123	0	-123
Staphopain A	*scpA*	SAUSA300_1890	143	1	-143

a-LAC wild-type

b- LAC protease-null mutant

c- total spectral counts identified for each protein

Supplemental Table 2. Individual fraction analysis of alterations in protein stability for secreted proteins upon deletion of extracellular proteases in *S. aureus* USA 300.

Protein	Gene	Accession Number	Size	Fold Change
Immunoglobulin-binding protein	*sbi*	SAUSA300_2364	50 kDa	9
Alpha-hemolysin	*hla*	SAUSA300_1058	36 kDa	8.88
Phenol-soluble modulin alpha 4 peptide	*psmα4*		2 kDa	5
Leukocidin	*lukB*	SAUSA300_1974	39 kDa	4.66
Triacylglycerol lipase	*lip*	SAUSA300_2603	77 kDa	4.6
Gamma-hemolysin component A	*hlgA*	SAUSA300_2365	35 kDa	4
Staphylococcal complement inhibitor	*scn*	SAUSA300_1919	13 kDa	3.66
Leukotoxin	*lukE*	SAUSA300_1769	35 kDa	3.66
Gamma-hemolysin component C	*hlgC*	SAUSA300_2366	36 kDa	3.33
Staphylococcal superantigen-like protein 7	*ssl7*	SAUSA300_0401	26 kDa	3
Extracellular matrix protein-binding protein	*emp*	SAUSA300_0774	38 kDa	3
Ear protein	*ear*	SAUSA300_0815	20 kDa	2.66
Triacylglycerol lipase precursor	*geh*	SAUSA300_0320	76 kDa	2.55
Panton-Valentine leukocidin	*lukS*	SAUSA300_1382	35 kDa	2.45
Autolysin	*atl*		137 kDa	2.3
Secretory antigen	*ssaA*	SAUSA300_2249	29 kDa	2
Extracellular matrix-binding protein	*ebh*	SAUSA300_1327	1123 kDa	2
Staphylococcal enterotoxin Q	*seq*	SAUSA300_0801	28 kDa	2
Leukocidin	*lukA*	SAUSA300_1975	40 kDa	2
Staphylococcal enterotoxin K	*sek*	SAUSA300_0800	28 kDa	2
Phenol-soluble modulin alpha 3 peptide	*psmα3*		3 kDa	2
Phenol-soluble modulin beta 1 peptide	*psmβ1*	SAUSA300_1067	4 kDa	1.75
Putative staphylococcal enterotoxin		SAUSA300_0370	23 kDa	1.66
Panton-Valentine leukocidin,	*lukF*	SAUSA300_1381	37 kDa	1.5

Supplemental Table 3. Collective alterations in protein abundance for surface proteins upon deletion of extracellular proteases in *S. aureus* USA 300.

Protein	Gene	Accession Number	WT[a,c]	M[b,c]	Fold Change
Fibronectin-binding protein B	*fnbB*	SAUSA300_1052	1	4	4
Fibronectin-binding protein A	*fnbA*	SAUSA300_2441	1	3	3
Enolase	*eno*	SAUSA300_0760	45	129	2.86
Staphylokinase	*sak*	SAUSA300_1922	16	40	2.5
Fibrinogen-binding protein	*efb*	SAUSA300_1055	9	21	2.33
Cell wall surface anchor family protein	*sasG*	SAUSA300_2436	33	74	2.24
Transferrin receptor	*tpn*	SAUSA300_0721	1	2	2
Putative lipoprotein		SAUSA300_0372	24	44	1.83
Clumping factor A	*clfA*	SAUSA300_0772	11	19	1.72
Iron-regulated surface determinant protein A	*isdA*	SAUSA300_1029	10	16	1.6

a-LAC wild-type
b- LAC protease-null mutant
c- total spectral counts identified for each protein

Supplemental Table 4. Individual fraction analysis of alterations in protein stability for surface proteins upon deletion of extracellular proteases in *S. aureus* USA 300.

Protein	Gene	Accession Number	Size	Fold Change
Clumping factor B	*clfB*	SAUSA300_2565	97kDa	8
Staphylokinase	*sak*	SAUSA300_1922	18 kDa	4.8
Fibrinogen-binding protein	*efb*	SAUSA300_1055	19 kDa	4.25
Fibronectin-binding protein B	*fnbB*	SAUSA300_1052	13 kDa	4
Enolase	*eno*	SAUSA300_0760	47 kDa	3.82
Iron-regulated surface determinant protein A	*isdA*	SAUSA300_1029	39 kDa	3.75
Fibronectin-binding protein A	*fnbA*	SAUSA300_2441	112 kDa	3
Cell wall surface anchor family protein	*sasG*	SAUSA300_2436	49 kDa	2.93
Putative lipoprotein		SAUSA300_0372	21 kDa	2.5
Putative surface protein		SAUSA300_0883	16 kDa	2.47
Probable transglycosylase	*isaA*	SAUSA300_2436	24 kDa	2.36
Immunodominant staphylococcal antigen B	*isaB*	SAUSA300_2573	19 kDa	2.2
Clumping factor A	*clfA*	SAUSA300_0772	97 kDa	2.16
Immunoglobulin G binding protein A	*spa*	SAUSA300_0113	56 kDa	2.1
Elastin-binding protein	*ebpS*	SAUSA300_1370	53 kDa	2
Transferrin receptor	*tpn*	SAUSA300_0721		2
Staphylocoagulase	*coa*	SAUSA300_0224	69 kDa	2

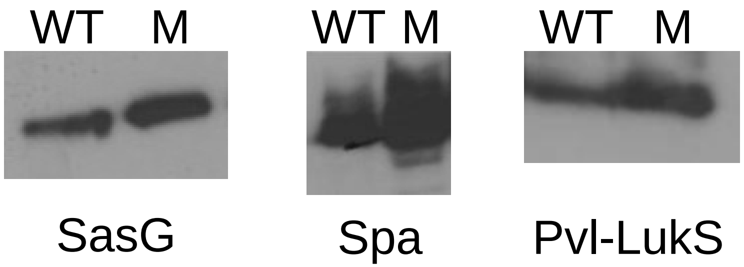

Supplemental Figure 3. Western blot analysis confirms increased abundance of select surface and secreted virulence factors upon exo-protease deletion. Secretome and surfactome samples were prepared in an identical manner as for our proteomic studies. Samples were standardized to total protein amounts, with equal concentrations loaded onto SDS-PAGE gels. These were then probed with antibodies specific to the relevant proteins to determine abundance in the LAC wild-type (WT) and protease null mutant (M).

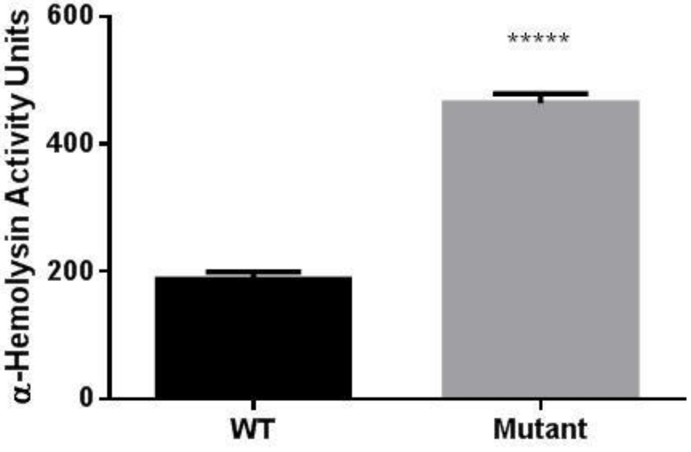

Supplemental Figure 4. Exoprotease deletion leads to increased α-hemolysin activity. α-hemolysin activity was assayed in 15h culture supernatants from wild-type and mutant strains using rabbit blood. Data presented is from four biological replicates. Error bars are shown ±SEM; ***** = $p<0.0001$.

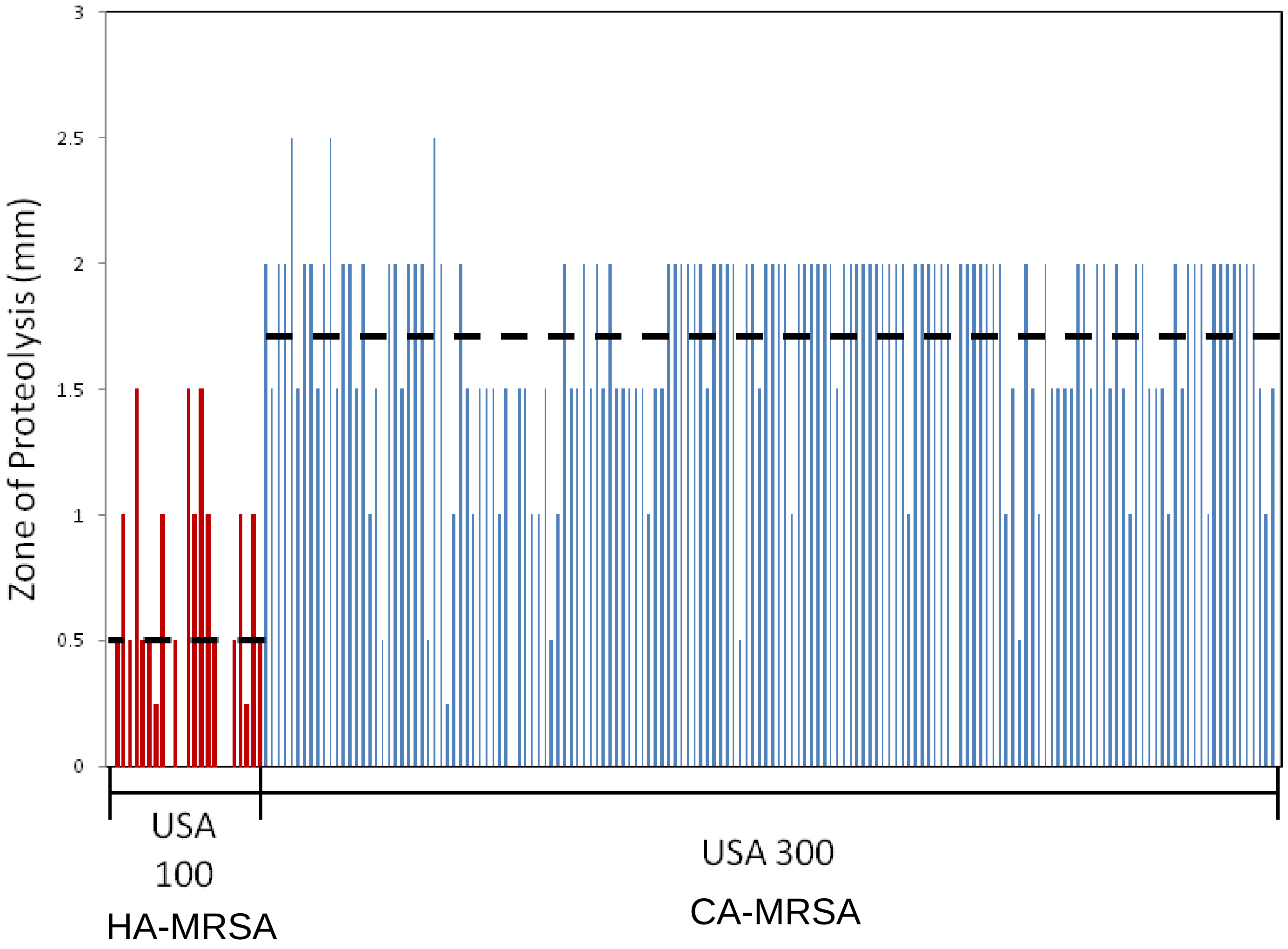

Supplemental Figure 5. Secreted proteolytic activity of clinical *S. aureus* isolates. Hospital-associated methicillin resistant *S. aureus* (HA-MRSA) strain USA100 (red) and community-associated methicillin resistant *S. aureus* (CA-MRSA) USA300 (blue) isolates from the Florida department of health state laboratory were streaked onto casein agar plates. Mean zones of proteolysis for each lineage are shown as a dashed black line (USA100 = 0.5mm; USA300 = 1.7mm).
